# Language proficiency and ethnic‐racial orientation among Latine mother–adolescent dyads

**DOI:** 10.1111/jora.12994

**Published:** 2024-06-26

**Authors:** Tahjanee Givens, Frances M. Lobo, Lisa Kiang, Gabriela L. Stein

**Affiliations:** ^1^ Department of Psychology Wake Forest University Winston‐Salem North Carolina USA; ^2^ Department of Human Development and Family Sciences University of Texas at Austin Austin Texas USA

**Keywords:** acculturation conflict, ethnic‐racial orientation, language

## Abstract

Language proficiencies have implications for how parents and children can communicate effectively and how culture and heritage can be transferred across generations. Previous research has sought to understand the relationship between parent language (mainstream, heritage) proficiencies and the ethnic‐racial orientation of their children, though prior studies have not investigated the relationship between child language proficiencies and parent ethnic‐racial orientation. This study examined the actor–partner effects of Latine mother–child dyads (*N* = 175; youth mean age = 12.86 years) regarding their proficiencies in English and Spanish and their Latine and White orientations. Our results revealed that youth Spanish language proficiency was positively linked to youth White orientation, and youth English proficiency was also positively associated with youth White orientation but only in instances when youth‐reported acculturation conflict was lower or average. There were two partner effects observed, with youth English proficiency positively relating to mother's White orientation and mothers' Spanish proficiency being negatively related to youth White orientation. Regarding Latine orientation, both English and Spanish were positively related to greater Latine orientation for both mothers and their children. However, at higher levels of mother‐reported acculturation conflict, higher mother English proficiency was related to lower youth Latine orientation. Overall, language proficiencies for Latine mothers and their children contribute to the development of bicultural orientations, though varying degrees of acculturation conflict can have differential impacts on these linkages.

## INTRODUCTION

The United States is a place of growing ethnic‐racial and cultural diversity. In recent decades, its very fabric has evolved and continues to change with the increasing visibility of ethnic‐racial groups. One central component of the diversity these groups provide is linguistic diversity. The 2022 American Community Survey estimated that nearly 22% of people in the United States speak a language other than English at home, and of those who speak a language other than English, they estimate the majority speak Spanish (U.S. Census Bureau, 2022). Existing research suggests that advantages can be found among bilingual speakers compared to monolinguals, such as higher levels of heritage language proficiency elevating levels of self‐esteem (Yu, 2015), more positive same‐race peer relationships (Kiang, 2008), and better socioemotional outcomes (Wang et al., 2022).

Given such correlates, it is important to note that, within Latine (see Appendix) households, there may exist varying levels of Spanish and English proficiency among family members and across the span of generations. For example, one study on the adjustment of two diasporic groups found generational differences between mothers and their children in heritage language proficiency (Aumann & Titzmann, 2018). Moreover, this gap in heritage language proficiency was larger than the gap between mothers and their children in host language proficiency (Aumann & Titzmann, 2018). This finding indicates that heritage language proficiency, in particular, might serve as a point of divergence between immigrant mothers and their children. Such intergenerational differences can encapsulate a mismatch between parents and children that extends beyond linguistic differences (Aumann & Titzmann, 2018), such as illustrating “acculturation gaps” in cultural values and overall heritage and mainstream orientations (Telzer, 2011). Indeed, understanding the development and retention of heritage and host languages demands one to consider the context in which these processes occur and what the implications of such processes might be. An extensive body of literature has examined bilingualism among Latine youth (Arredondo & Rosado, 2016; Mu, 2015; Oh & Fuligni, 2010; Phinney et al., 2001; Tran, 2010). However, research has yet to consider nuanced and transactional associations between how youth linguistic proficiencies (heritage, mainstream) and cultural orientations (heritage, mainstream) might be linked with those of their parents and vice versa.

The present study uses a dyadic framework to investigate the intersectional relations between parent–child Spanish and English proficiencies and Latine and White orientations. Although prior work has sought to understand how parents' language proficiencies could predict a child's language proficiencies and/or ethnic‐racial orientations, a gap remains in using the child's language proficiencies or ethnic‐racial orientations as predictors of parents' outcomes. Hence, our approach enlists actor–partner interdependence models to determine how the language proficiencies of the mother may relate to the orientations of the child and how the child's language proficiencies may relate to the orientations of the mother.

### Importance of ethnic‐racial orientation

Beyond the linguistic diversity evident in a growing number of Spanish speakers in the United States, the Latine population reflects diverse traditions, practices, and values. Yet, adolescents from minoritized backgrounds, particularly those from immigrant families, uniformly face the challenge of both acculturation and enculturation (Ferguson et al., 2012; Schwartz et al., 2010). Acculturation is a developmental process whereby one gains values, traditions, practices, and sometimes language from a culture beyond one's heritage culture, such as the new culture one encounters after immigration. Enculturation, another developmental process, refers to the learning of one's heritage language, values, and practices. Previous models have identified four patterns for enculturation and acculturation, from integrating (expressing high levels of both heritage and host cultures) to feeling marginalized (expressing low levels of both heritage and host cultures; Berry et al., 2006). Such models of acculturation provide a theoretical basis for understanding when two cultural identities can be dually fostered to create a *bicultural* identity (Comănaru et al., 2018). Similar to the advantages observed for bilingual speakers, bicultural adolescents generally display better psychological and sociocultural outcomes than those who solely identify with either their heritage or mainstream identity (Berry et al., 2006).

Cultural orientation reflects the degree to which an individual endorses culturally specific attitudes, behaviors, and emotions. Cultural orientation and identity are both elements of acculturation and enculturation though orientation and identity are each unique constructs. For example, previous work distinguishing ethnic‐racial orientation from identity has found that perceived parental acculturation is related to a young adult's culture‐specific consumption behaviors (an acculturative behavior), but not to the young adult's ethnic identity (Xu et al., 2004). Hence, parental influences occurring in the development of ethnic‐racial orientation (versus identity) through processes of enculturation and acculturation might be particularly salient for adolescents. Although previous studies have connected parental heritage language proficiency to their and their child's ethnic‐racial orientations, we have not found a study that adopts the actor–partner interdependence model to understand the reverse, or how children's language proficiencies might shape the ethnic‐racial orientations of their parents. The present study addresses these gaps in the literature by focusing on dyadic associations between parents' and children's language proficiencies (Spanish, English) and ethnic‐racial orientations (Latine, White).

### Associations between language proficiency and ethnic‐racial orientation

Language is a core feature of human development and progresses within the specific environmental context of an individual. A substantial body of literature has linked heritage language proficiency to ethnic identity and orientation among youth and adolescents (Park, 2022; Phinney et al., 2001; Wang et al., 2022; Yu, 2015). A meta‐analysis consisting of 43 datasets across 18 studies generally found a positive trend between heritage language proficiency and ethnic identity, a finding that withstood multiple ethnic groups (Mu, 2015). Similarly, research has shown connections between host language proficiency levels and sense of belonging and identification with the host culture (Amit & Bar‐Lev, 2015). Language, therefore, appears to serve as a conduit through which adolescents and adults experience their own culture and acculturate to the host culture.

Yet, there are differences among ethnic‐racial groups in their retention of heritage language (Tran, 2010), sociocultural and psychological adaptations (Vedder & Virta, 2005), and the associations between language and identity (Phinney et al., 2001). Previous studies focusing on immigrant youth in the United States have additionally found differences between Asian American and Latine youth in heritage language proficiencies (Oh & Fuligni, 2010) and within‐group differences in the Latine community in terms of heritage language maintenance (Tran, 2010). Therefore, various factors can influence both (1) parent and child language proficiencies resulting in intergenerational differences between mothers and their children as well as (2) the associations between language proficiencies and ethnic‐racial orientations.

Building upon these ideas, the *integrative model* encourages researchers to include broader sociocultural contexts and consider systems of oppression (e.g., racism, classism) in understanding the development of minoritized youth (García‐Coll et al., 1996). For example, language development occurs within a unique sociopolitical context for each ethnic‐racial group, which can also influence the family as a micro‐level structure. The simultaneity of the macro‐ and micro‐level structures, in turn, has implications for parents' and children's language proficiencies as well as their orientations toward the mainstream or their heritage group. The present study uses the distinction of “White” culture as a measure of acculturation to account for the unique ethnic‐racial context of the United States. Previous research has demonstrated that tridimensional acculturation and enculturation occur for immigrant adolescents in the American context (e.g., European‐American, African‐American), demonstrating the importance of specificity in articulating what the assumed mainstream culture is (Ferguson et al., 2012).

Parental heritage language proficiency tends to relate to that of the child (Aumann & Titzmann, 2018; Chen & Kang, 2019); however, parents of bilingual children nevertheless face the dilemma of wanting to promote heritage language retention within their children while simultaneously being acutely aware of the need to acquire the dominant language of the country (Curdt‐Christiansen, 2009). Therefore, parents may also feel the need to strike some form of balance, which could include more heritage language than host language or vice versa, for themselves. Although previous studies have reflected that there is a continued change in ethnic‐racial orientation for adults as they acculturate and that parental cultural orientation develops alongside their child's (Wang‐Schweig & Miller, 2021), unanswered questions remain in how the child may be influencing their parent's process of acculturation. Therefore, understanding children's outcomes requires simultaneously an understanding of parents' outcomes and what influences this dual relationship might have on each of the partners involved.

### Moderation by acculturation conflict

In light of the dynamic interactions between parent–child language proficiencies and ethnic‐racial orientation, it is also important to consider specific features of the relationship context. For example, children's heritage language proficiency has been shown to be positively associated with parent–child factors such as the child's perceived warmth of the mother (Wang et al., 2022) and the overall quality of the parent–adolescent relationship (Oh & Fuligni, 2010). Consistent with this work and additionally embedded in culturally relevant constructs is the possible generational discrepancy between parents and children in their overall levels of acculturation. Among immigrant families, this “acculturation gap” can sometimes lead to conflict or distress arising from a perceived difference between parents and children in their values, relationship, and identifications (Telzer, 2011). Although the acculturation gap between parents and adolescents is often viewed through a deficit lens that assumes the parent would be less acculturated than the adolescent in the host culture and, therefore, youth experience negative outcomes, emerging models point to a range of ways that an acculturation gap can happen, such as when the parent is more acculturated than the adolescent (Telzer, 2011). Although acculturation gaps do not have inherently negative outcomes, parents and adolescents could still encounter acculturation conflict, which specifically refers to the inconsistencies between the parent and child when adapting to a new culture and the challenges that can be faced during the process (Huq et al., 2016; Portes & Rumbaut, 2001). Larger gaps in acculturation can contribute to familial conflict between parent and child and can be a source of tension, having negative impacts on both the parent and child (Kiang et al., 2017; Zhang & Kong, 2022).

The impact of greater acculturation conflict extends to negative outcomes such as poorer child adjustment (Chen et al., 2014) and depressive symptoms (Huq et al., 2016). Acculturation conflict has been shown to be associated with a host of factors, including greater gaps in heritage language proficiency (Portes & Hao, 2002) and lower child private regard—a key aspect of ethnic‐racial identity (Huq et al., 2016). The effects of perceived acculturation conflict have been incorporated in actor–partner analyses where acculturation levels of the parent and the adolescent were utilized as predictors (Wang‐Schweig & Miller, 2021). Given evidence from prior work illustrating the high relational interdependence involved in acculturation conflict and inherent links between acculturation and enculturation processes and culturally relevant behaviors such as language proficiency, we explored the role of acculturation conflict as a moderator of the expected associations between language proficiency and ethnic‐racial orientation. More specifically, we expected high levels of perceived conflict to reflect a greater disconnect between parent–child dyads, thereby weakening any actor–partner effects that might be found. Our particular focus on mothers is due to prior work suggesting that they tend to be the primary caregivers among Latine families and could also have relatively stronger influences on youth development (e.g., self‐esteem) compared to fathers (Bámaca et al., 2005; Galvan et al., 2022).

### The current study

The present study seeks to understand the dynamic and interdependent environment in which language proficiencies (Spanish, English) and ethnic‐racial orientation (Latine, White) develop within mother–child relationships. Our approach offers a unique contribution by using dyadic analyses that examine not only actor associations (e.g., mothers' language proficiency as related to their own ethnic‐racial orientation) but also partner associations (e.g., mothers' language proficiency as related to their child's ethnic‐racial orientation). Moreover, our actor–partner models not only capture mother‐to‐child associations but also those of child‐to‐mother. We incorporate multiple dimensions of the immigrant experience through critical, culturally relevant processes related to heritage (e.g., Spanish proficiency, Latine orientation) and host or mainstream characteristics (e.g., English proficiency, White orientation). In examining these actor and partner associations, we also explore the possible moderating effect of acculturation conflict, as reported independently by mothers and adolescents. Taken together, the present study recognizes and aims to shed light on the interdependent nature of developing a sense of ethnic‐racial orientation, ultimately providing new knowledge on how such processes might be best promoted among both parents and children from immigrant backgrounds.

## METHODS

### Participants

Participants were 175 mother–adolescent dyads from Latine backgrounds. The mean age of youth was 12.86 years (SD = .68; range = 10.33–15.23). Just over half of the youth were female (51.4%) and 86.9% were U.S.‐born. For those not U.S.‐born, the average age of immigration was 4.25 years old. Almost all mothers were foreign‐born (98%), with an average length of time in the United States of 15.67 years (SD = 4.61). The majority of families had Mexican heritage (89%). Other countries of origin included Columbia, Dominican Republic, Ecuador, El Salvador, Guatemala, Honduras, Nicaragua, and Puerto Rico. Mean family annual income was $23,020 (SD = $12,390), with an average of 4.72 (SD = 1.10) people living off that income. In terms of education, 45% of mothers reported receiving less than an 8th‐grade education, 47% ranged from 8th grade to high school or GED, and 8% reported having completed or having some post‐high school education.

### Procedures

Families were largely recruited from two public middle schools in a semi‐rural, emerging immigrant region of the Southeastern United States. Per IRB guidelines and in collaboration with the schools, flyers and letters about the study were mailed home to students. Using call lists of 7th and 8th graders who were identified as Latine by the school, project staff recruited interested and eligible families based on the following criteria: (a) both biological parents were also from Latine backgrounds, (b) the mother was the resident caregiver of the participating child, and (c) youth ranged between 11 and 14 years of age. An additional recruitment phase included door‐to‐door home visits to target families who could not be reached via phone. This process was repeated twice yearly, with the recruitment of incoming 7th graders and 8th graders in the schools.

Across recruitment strategies, the current study attempted to reach a total of 597 families via phone or door‐to‐door. Of these, 16 families had moved (3%) and 217 were not located (e.g., disconnected numbers, families not home; 36%). Of those who were successfully contacted (*n* = 364), 47 were not eligible (13%), 125 declined (34%), 16 consented but did not complete interviews (4%), and 176 families consented and completed interviews (48%). Upon study enrollment, research assistants (including at least one Spanish‐speaking RA) visited homes to administer consent and questionnaires, which took about 60–90 minutes to complete. Materials were available in Spanish and English and administered according to the participant's preference. Following completion, youth were given a $10 gift card and mothers were given a $20 gift card.

### Measures

#### Language proficiency

English and Spanish proficiency were each measured using three items adapted from the Asian American Multidimensional Acculturation Scale (AAMAS; Chung et al., 2004). Specifically, the language items from the AAMAS were modified to assess proficiency in speaking, reading/writing, and understanding English and Spanish, separately. Items were scored on a 1 (*Not Very Well*) to 6 (*Very Well*) scale and averaged to compute language proficiency scores. The internal consistencies were for both mothers and youth for English (mothers: α = .89; adolescents: α = .78) and Spanish (mothers: α = .79; adolescents: α = .79).

#### Latine and White orientation

The two cultural orientation dimensions were also measured using adapted items from the AAMAS. For the White orientation scale, participants were asked about their identity, behaviors, and knowledge about White mainstream culture. There were 12 items scored on a 1 (*Not Very Much*) to 6 (*Very Much*) scale. Sample items include, “How often to you listen to music or look at movies and magazines from…How knowledgeable are you about the culture and traditions of…How much do you identify with…How much do you feel you have in common with…” with an ending stem of “White mainstream groups?” For the Latine orientation scale, participants responded to identical items that included ending stems of “Your own Latino culture of origin (e.g., Mexican)?” and “Other Latino groups in America?” These two latter sets of items were combined to reflect the participant's identity, behaviors, and knowledge about Latine culture (24 total items). Item responses were averaged together to yield an overall score for White or Latine orientation. Internal consistencies were the following for White orientation (mothers: α = .90; adolescents: α = .89) and Latine orientation (mothers: α = .89; adolescents: α = .88).

#### Acculturation conflict

The Acculturation Gap Conflict Inventory (Basáñez et al., 2014) assessed the degree to which participants reported having disagreements about their preference for the heritage or host culture. Parents responded to 9 items such as “Sometimes I tell him or her to speak Spanish instead of English” or “I think he or she would rather be more American if given the chance” on a scale from 1 (*Strongly Disagree*) to 7 (*Strongly Agree*). Youth responded to 7 items such as “I get upset at my parents because they do not know American ways of doing things” and “My parents complain that I act too American” on a scale from 1 (*Strongly Disagree*) to 5 (*Strongly Agree*). Items were averaged together to yield total scores for mother‐reported (α = .79) and youth‐reported (α = .91) acculturation conflict over a preferred culture.

### Data analytic plan

Actor–partner interdependence models (APIMs; Kashy & Kenny, 2000) allow us to consider the interdependence between mother and youth when exploring dyadic relationships. In the present study, we tested whether one's own English or Spanish proficiency predicted their own reported White or Latine orientation (i.e., actor effects) or their partner's reported White or Latine orientation (i.e., partner effects). Additionally, we tested whether maternal and youth reports of acculturation conflict moderated these actor and partner effects via 8 separate APIMs. We mean‐centered and standardized all variables and then estimated the APIMs using the *nlme* package (Pinheiro et al., 2022) in RStudio version 1.183 (RStudio Team, 2009–2019). To probe significant interaction effects, we tested the effects of language proficiency on ethnic‐racial orientation at values of the moderator that were lower (i.e., one standard deviation below the mean; −1SD) and higher (i.e., one standard deviation above the mean; +1SD) using the *interactions* package (Long, 2019) in RStudio version 1.183 (RStudio Team, 2009–2019).

We only included families with complete data in any given model; there were 9 to 27 families missing data in any given analysis. Twenty‐eight families were missing data across all the study variables. These families did not differ from families with complete data in relation to family income, youth gender and grade, and maternal age, education, marital status, and number of years in the United States. However, families with complete data reported older adolescents on average (*M* = 12.93) than families with incomplete data (*M* = 12.66), *t*(42.24) = 2.05, *p* < .05.

## RESULTS

### Preliminary analyses

Mean and bivariate correlations among the primary study variables are shown in Table 1. Maternal English proficiency exhibited moderate positive correlations with maternal White and Latine orientation, whereas youth English proficiency displayed weak to moderate positive correlations with maternal White orientation and both youth White and Latine orientation. Maternal Spanish proficiency showed a positive association with maternal Latine orientation, and youth Spanish proficiency was positively correlated with youth White and Latine orientation. Mothers and children reported generally similar mean levels of cultural orientation, with White orientation averaging around the midpoint of the scale and Latine orientation falling slightly higher. In contrast, mothers reported quite low levels of English proficiency (*M* = 2.30 on a 6‐point scale) but higher Spanish proficiency, whereas children reported much higher English proficiency (*M* = 5.77) and moderate levels of Spanish proficiency.

**TABLE 1:** Descriptive statistics and correlations for study variables.

Variable	1	2	3	4	5	6	7	8	9	10	11	12	13	*n*	*m*	SD
1. Maternal age	1													173	42.25	8.01
2. Youth age	.03	1												173	12.89	0.78
3. Maternal years in the US	.24**	.02	1											172	15.67	4.61
4. Youth gender	−.02	.03	.10	1										174	0.48	0.50
5. English proficiency (P)	−.06	.06	.18*	.12	1									175	2.30	1.28
6. English proficiency (Y)	.03	−.04	.17*	.14^T^	.18	1								175	4.55	0.60
7. Spanish proficiency (P)	.12	−.20*	−.04	.05	.07	.14^T^	1							175	5.77	0.60
8. Spanish proficiency (Y)	−.01	−.11	‐.13^T^	−.06	.04	.17*	.22**	1						175	3.73	0.97
9. AC (P)	< .001	.07	.08	−.04	−.12	.13^T^	.06	−.06	1					174	3.05	1.33
10. AC (Y)	−.07	.03	−.16*	.01	−.06	−.09	.05	−.16*	.17*	1				155	2.30	1.01
11. White orientation (P)	−.12	−.001	.28***	< .001	.43***	.25***	.02	.07	.08	.03	1			175	3.31	1.04
12. White orientation (Y)	.09	.13^T^	.07	−.01	.08	.22**	−.11	.19*	.03	−.04	.22**	1		175	3.62	0.74
13. Ethnic orientation (P)	< .001	−.04	.01	.09	.24**	.08	.22**	.13^T^	.06	.02	.49***	.12	1	175	4.30	0.77
14. Ethnic orientation (Y)	−.04	−.03	−.01	−.09	−.02	.18*	.02	.42***	−.15*	−.21*	.06	.48***	.02	175	4.08	0.52

*Note*: *N* = 175.

Abbreviations: AC, acculturation conflict; *M*, mean; P, mother‐report; Y, youth‐report; SD, standard deviation.

^T^ *p* < .10;

* *p* < .05;

** *p* < .01;

*** *p* < .001.

### White orientation APIMs

We used four APIMs to examine the moderating effects of mother‐ and youth‐reported acculturation conflict on the relations between English or Spanish proficiency and White orientation, controlling for maternal age, youth age, maternal years in the United States, and youth gender. Across all models (see Table 2), there was a positive youth actor effect indicating that youth‐reported English or Spanish proficiency was positively associated with youth White orientation. In other words, greater proficiency in either language was related to youth having a higher White orientation. Interestingly, maternal Spanish proficiency was negatively related to youth White orientation. Also, mother‐ and youth‐reported English proficiency were positively associated with maternal White orientation; however, this effect was not observed in the Spanish proficiency models. In all models, being younger or having spent more years in the United States was related to higher perceived White orientation among mothers.

**TABLE 2:** White orientation and acculturation conflict (AC) actor–partner interdependence models.

Variable	English model with mother‐reported AC		English model with youth‐reported AC		Spanish model with mother‐reported AC		Spanish model with youth‐reported AC	
	*b*	SE	*b*	SE	*B*	SE	*b*	SE
Predicting maternal White orientation								
Intercept	.08	.10	.11	.10	.02	.10	.03	.11
Language proficiency (mother)	.35***	.08	.32***	.08	.05	.08	.07	.08
Language proficiency (youth)	.15*	.07	.18*	.07	.07	.08	.08	.08
AC	.11	.07	.10	.07	.08	.07	.11	.08
Lang prof. (mother) × AC	.08	.08	−.04	.08	−.001	.09	.05	.09
Lang prof. (youth) × AC	−.01	.07	−.001	.07	−.0005	.08	−.09	.08
Maternal age	−.23*	.08	−.22*	.08	−.31***	.09	−.29**	.09
Youth age	−.03	.07	−.05	.07	.01	.08	−.02	.08
Maternal years in the US	.26***	.07	.27***	.07	.37***	.08	.40***	.08
Youth gender	−.15	.14	−.18	.15	−.08	.15	−.09	.16
Predicting youth White orientation								
Intercept	.04	.11	.02	.11	−.01	.11	−.07	.11
Language proficiency (mother)	.02	.09	.01	.09	−.13	.08	−.18*	.09
Language proficiency (youth)	.22*	.08	.21*	.08	.24**	.08	.28**	.09
AC	−.01	.08	−.04	.08	.04	.08	.04	.08
Lang prof. (mother) × AC	−.06	.10	−.14	.09	.01	.09	−.14	.10
Lang prof. (youth) × AC	.05	.08	−.21*	.08	.05	.08	−.09	.09
Maternal age	.05	.10	.05	.10	.07	.09	.08	.10
Youth age	.13	.08	.13	.08	.11	.08	.12	.08
Maternal years in the US	.05	.08	.03	.08	.08	.08	.09	.08
Youth gender	−.12	.16	−.15	.16	−.01	.16	.005	.16

*Note*: Youth gender coded as 0 = female, 1 = male.

Abbreviations: b, standardized regression coefficient; SE, standard error.

* *p* < .05;

** *p* < .01;

*** *p* < .001.

**FIGURE 1:**
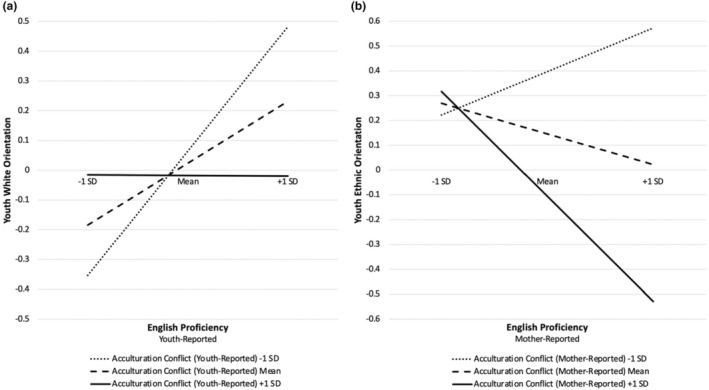
Acculturation Conflict Moderates the Effect of Language Proficiency on Ethnic Identity. Examining the moderating effects of acculturation conflict on the relations (a) between youth English proficiency and youth White orientation and (b) between maternal English proficiency and youth ethnic orientation at one standard deviation above the mean (+1SD) and one standard deviation below the mean (−1SD).

We observed one moderation effect across the four models. Youth‐reported acculturation conflict moderated the association between youth‐reported English proficiency and youth‐reported White orientation. Post‐hoc analyses (see Figure 1a) revealed that there was a positive link between youth‐reported English proficiency and youth‐reported White orientation at lower levels of acculturation conflict (−1 SD), *b* = .42, SE = .11, *p* < .001, and mean levels, *b* = .20, SE = .08, *p* = .01. However, there was no association at higher levels (+1 SD).

### Latine orientation APIMs

We conducted four APIMs examining the moderating effects of mother‐ and youth‐reported acculturation conflict on the relations between language proficiency (English or Spanish) and Latine orientation, controlling for maternal age, youth age, maternal years in the United States, and youth gender. The results are reported in Table 3. Across all models, we found evidence for moderate positive actor effects but no partner effects (i.e., one's own English or Spanish proficiency was positively related to their own reported Latine orientation, but not their partner's). Unsurprisingly, mother‐ and youth‐reported acculturation conflict were also negatively related to youth‐reported Latine orientation, indicating that higher perceived acculturation conflict was associated with lower youth Latine orientation. In the model examining English proficiency and mother‐reported acculturation conflict, youth gender was also negatively related to youth‐reported Latine orientation, such that being male was related to lower youth Latine orientation; however, this finding was not observed in the other models.

**TABLE 3:** Ethnic orientation and acculturation conflict (AC) actor–partner interdependence models.

Variable	English model with mother‐reported AC		English model with youth‐reported AC		Spanish model with mother‐reported AC		Spanish model with youth‐reported AC	
	*b*	SE	*b*	SE	*B*	SE	*b*	SE
Predicting maternal ethnic orientation								
Intercept	−.07	.11	−.04	.11	−.13	.11	−.08	.11
Language proficiency (mother)	.28**	.09	.20*	.09	.21*	.08	.25**	.09
Language proficiency (youth)	.002	.08	.06	.08	.10	.08	.07	.08
AC	.11	.08	.03	.08	.06	.08	.03	.08
Lang prof. (mother) × AC	.004	.09	−.10	.09	.14	.09	.09	.09
Lang prof. (youth) × AC	−.12	.08	−.05	.08	−.003	.08	−.06	.09
Maternal age	.05	.09	.05	.10	−.04	.09	−.04	.10
Youth age	−.06	.08	−.06	.08	.02	.08	.01	.08
Maternal years in the US	−.08	.08	−.04	.08	.04	.08	.05	.08
Youth gender	.19	.16	.14	.16	.23	.16	.19	.16
Predicting youth ethnic orientation								
Intercept	.15	.10	.13	.12	.12	.10	.07	.11
Language proficiency (mother)	−.12	.08	−.03	.09	−.02	.08	−.07	.08
Language proficiency (youth)	.24**	.08	.19*	.08	.42***	.07	.45***	.08
AC	−.25**	.08	−.24**	.08	−.13^T^	.07	−.16*	.08
Lang prof. (mother) × AC	−.30**	.09	−.08	.09	−.01	.08	−.10	.09
Lang prof. (youth) × AC	.001	.07	−.06	.08	.06	.08	.02	.08
Maternal age	−.13	.09	−.17^T^	.10	−.11	.09	−.14	.09
Youth age	−.02	.07	−.02	.08	−.004	.07	.001	.08
Maternal years in the US	.06	.08	−.005	.09	.09	.07	.07	.08
Youth gender	−.36*	.15	−.29^T^	.17	−.23	.14	−.17	.15

*Note*: Youth gender coded as 0 = female, 1 = male.

Abbreviations: *b*, standardized regression coefficient; SE, standard error.

^T^ *p* < .10;

* *p* < .05;

** *p* < .01;

*** *p* < .001.

One moderation effect was also found across these four models. Mother‐reported acculturation conflict moderated the relation between maternal English proficiency and youth‐reported Latine orientation. Post‐hoc analyses (see Figure 1b) revealed that at higher levels of acculturation conflict (+1 SD), higher maternal English proficiency was related to lower youth‐reported Latine orientation, *b* = −.43, SE = .13, *p* < .001, but there was no association among these variables at mean and lower levels (−1 SD) of acculturation conflict.

## DISCUSSION

The present study explored the actor and partner associations between language proficiency and ethnic‐racial orientations among Latine mother–child dyads. Language is essential to how parents and children can effectively communicate about their heritage as well as integrate new cultures into their understanding of themselves in various sociocultural contexts (Park, 2022; Phinney et al., 2001; Wang et al., 2022; Yu, 2015). The findings from this study indicate that mainstream language (English) and heritage language (Spanish) have effects on ethnic orientation and White orientation for both mothers and children. Additionally, this study found actor effects for both mother and child (i.e., one's own language proficiencies linked to their ethnic‐racial orientations) and partner effects (i.e., mother's language proficiency predicted their child's ethnic‐racial orientations and vice versa). This study also considered the importance of maternal age, the number of years the mother reported living in the United States, and the gender of the child in understanding the complexity of the dyadic relationship. The perspective of acculturation and enculturation as ongoing processes for both parents and children in Latine families can also help inform our conception of biculturality and what other factors may contribute to identity development processes for Latine mothers and children.

### White orientation

The results from this study illuminate multiple pathways to White orientation among Latine adolescents and their mothers. We observed actor effects of both Spanish and English, with both languages linking to greater White orientation for adolescents. Therefore, adolescents who had higher proficiencies in English, as well as those who had higher proficiency in Spanish, tended to have a greater White orientation. Unsurprisingly, English for mothers was linked to greater White (mainstream)‐oriented behaviors, such as listening to music from the mainstream culture. This pattern of results connecting language and acculturative behaviors is consistent with previous research that has shown the linkages between mainstream language use and mainstream cultural orientation (Zea et al., 2003). This study extends previous literature on the roles of language in acculturative processes by demonstrating that, for children, both mainstream and heritage language are important pathways to mainstream cultural orientation, while for mothers, mainstream language, in particular, is essential to their mainstream orientation development.

However, the positive relation between adolescent English proficiency and adolescent White orientation was no longer significant except when youth reported lower or mean levels of acculturation conflict. That is, a child's English proficiency was positively related to their White orientation only when their reported conflict stemming from cultural differences with their mother was lower or average. A potential explanation for this result is that as conflict with mothers relating to culture increases, adolescents' exploration of the mainstream culture may be curtailed as a result. In addition, in terms of adolescents' mainstream orientation, partner effects revealed that maternal Spanish language proficiency predicted lower adolescent White orientation across varying levels of acculturation conflict perhaps because greater exposure and access to parental Spanish language use limits youth engagement with White cultural behaviors and traditions. Whereas past research has shown the influence of familial relations on the association between heritage language and mental health outcomes (Huq et al., 2016; Kilpi‐Jakonen & Kwon, 2023), this study reveals the effect of language (mainstream, heritage) on ethnic‐racial orientations as well as the moderating role of familial relations in dyadic outcomes. The patterns suggest that familial dynamics between mothers and their children with lower levels of acculturation conflict might be more conducive to acculturation processes that further strengthen the connection between English proficiency and behaviors adaptive to White mainstream culture.

With respect to mothers' outcomes, the findings present evidence that children's mainstream language proficiency is also related to the mainstream‐oriented behaviors of their parents. The results revealed a partner effect with adolescent English proficiency predicting mothers' White orientation. Our findings suggest that adolescent‐driven factors help to explain in part their mother's adoption of White mainstream behaviors. This underscores the importance of considering adolescents as sources of ethnic‐racial socialization for their parents, particularly in the context of families that have experienced recent migration. Building on existing research showing evidence for adolescent‐directed racial‐ethnic socialization messages (Patel et al., 2023), this study raises questions about how language can fit into future models of adolescent‐directed socialization behaviors. Ultimately, this finding provides new insight into how the acculturation process in mother–child dyadic relationships can also reflect a directional dynamic from child to parent. Given the importance of context of migration (Aumann & Titzmann, 2018), generational differences (Oh & Fuligni, 2010), and familial closeness (Wang et al., 2022) for Latine families' heritage and ethnic identity development, research should include youth as part of the integrative context through which they seek to understand Latine parents' outcomes.

### Latine orientation

Consistent with prior research findings (Mu, 2015; Phinney et al., 2001; Wang et al., 2022), we observed positive associations between language proficiency and (Latine) ethnic orientation for Latine mothers and youth. For both English and Spanish proficiencies, there were strong actor effects in mothers and youth relating language proficiency to Latine orientation (i.e., there was a positive association between youth's own language proficiency and youth's Latine orientation and mother's own language proficiency and mother's Latine orientation). The positive relationship between English language proficiency and Latine orientation suggests that the higher the English language proficiency of the adolescent, the more likely they are to seek out the history, traditions, and practices related to their ethnic group. One interpretation of these findings linking English and ethnic orientation highlights that, in the context of the United States where Latines continue to experience marginalization, Latine mothers and their adolescents might be compelled to establish stronger connections with their ethnic‐racial heritage group as a source of resilience. In addition to fostering a sense of belonging, understanding and learning more about one's ethnic group appeared to be facilitated by proficiencies in both mainstream and heritage languages. Taken together, dual linguistic pathways via both English and Spanish may serve important purposes for Latine orientation for both mothers and their children.

Existing literature highlights heritage language proficiency as contributing to ethnic‐racial identity (Phinney et al., 2001; Yu, 2015). Our findings build on this to suggest an association linking heritage language proficiency with ethnic orientation, which reinforces the importance of developing heritage language proficiency and considering the influence this may have on the cultural behaviors one may exhibit as a part of their ethnic group.

More specifically, findings revealed that the higher the language proficiency of the mother in English, the lower the youth Latine orientation tended to be, but only when the mother's perception of acculturation conflict between her and her adolescent was high. While existing studies have shown the negative outcomes that can be attributed to acculturation conflict, particularly for Latine youth (Huq et al., 2016), the present study imparts a further consideration of how acculturation conflict may play a role in the ethnic‐group‐oriented behaviors of both Latine mothers and their children.

### Covariates

Within this study, we explored several covariate effects, including maternal age, time spent in the United States, and gender. Firstly, maternal age was negatively related to mothers' White orientation, meaning that younger mothers were more likely to report behaviors oriented toward the (White) mainstream culture than those who were older. It may be the case that younger mothers adopt more behaviors of the mainstream culture due to closer proximity to a developmental period of adolescence when belonging is essential and when assimilation processes appear to be more easily facilitated (Portes & Rumbaut, 2001). Similarly, mothers who have spent more time in the United States were more likely to report higher White orientation than those who have spent less time in the United States. This finding implies that longer exposure to a mainstream culture may be associated with a greater tendency to adopt behaviors from that mainstream culture. Hence, acculturation, as a developmental process, might be particularly influential when starting at a younger age and lasting over a longer period of time. These findings highlight the importance of age as a factor in acculturation; however, they emphasize the need to understand how age and time spent in the United States can influence the acculturative behaviors of parents in addition to their children.

For Latine adolescents, differences in Latine orientation were observed when taking into consideration gender. Latine male adolescents generally reported lower Latine orientation than their female Latine adolescent counterparts. We infer from this finding that there may be gendered aspects of ethnic‐racial socialization that led to our observed difference in female and male adolescents' adoption of behaviors related to their ethnic group. This finding is unsurprising when accounting for previous studies that have shown female Latine adolescents tend to retain heritage language proficiency at higher rates (Chen & Kang, 2019) and exhibit higher regard for their ethnic group (Huq et al., 2016) than male Latine adolescents. Nevertheless, these findings raise questions about what potential mediators may be leading us to observe gender differences among Latine adolescents and their relationship to their ethnic group (e.g., socialization messages). Future studies should further explore the mechanisms behind these gender‐based differences.

### Limitations and future directions

Though this study was innovative in using actor–partner interdependence models to understand the complex, dyadic relationship between language proficiencies and ethnic‐racial orientations, there remain a few limitations. First, the data collection in this study was limited to Latine mother–child dyads. Existing research has shown possible differences between Latine mothers and fathers in how they communicate about their culture with their children and transmit their heritage to subsequent generations (Zhang et al., 2019). By expanding research to Latine father–child dyads, new actor–partner patterns may be revealed around language proficiencies and White (mainstream) and Latine (heritage) orientations. Second, due to the relative homogeneity of our sample in terms of language proficiencies, this study would benefit from the inclusion of varying levels of mother Spanish and child English proficiencies. Few mothers reported being low in Spanish proficiency, whereas few children reported being low in English proficiency. While this study nevertheless bore interesting and meaningful results, by including a broader variety of language proficiencies, it may better capture a varied array of acculturative and enculturative dynamics (child more enculturated than parent, parent more acculturated than child) that remain understudied. Additionally, this study is limited in that it was not able to fully explore cultural variations among Latine mothers and their children. The ethnic‐racial group “Latine” is a very heterogenous group comprised of many cultural backgrounds. We did conduct post‐hoc sensitivity analyses that controlled for mothers' country of origin using a binary variable (see Tables S1 and S2, results were not substantively changed), but we were limited in our exploration since the majority of mothers (89%) were of Mexican‐origin. Hence, research that more systematically aims to understand the diverse cultural experiences of families who identify as Latine is needed to better uncover nuances related to acculturation and enculturation behaviors. Lastly, the present study did not explore the unique dimensions of the behaviors associated with the White and Latine orientations respectively. In 2017, Choi et al. found that various types of acculturative behaviors could have differential impacts on the outcomes of Korean American youth. Future studies should investigate what specific acculturative and enculturative behaviors relate uniquely to language and whether the actor and partner findings withstand a deeper analysis of specific behaviors.

#### Implications

Despite these limitations, the present study has enriched our understanding of the relationship between language proficiency and ethnic‐racial orientations by more specifically considering the role of independent and interdependent processes through the avenue of language for Latine youth and their mothers. Broadly, these findings support that attention should be focused on the simultaneous support of mainstream and heritage language development, though extra care should be centered on heritage language development for youth. With the apparent link between ethnic‐racial identity and mental health (for a review see Balidemaj & Small, 2019), this study can provide further insight into what factors may influence ethnic‐racial identity, including perceived acculturation conflict. By improving our understanding of Latine mother–child relationships, future interventions can explore ways to minimize acculturation conflict that supports bicultural identity development. We have also shown that future research and interventionists should consider the ways in which Latine youth can influence the outcomes of their mothers, particularly when it relates to acculturative behaviors.

## CONCLUSION

Though a great deal of literature exists on the processes of acculturation and enculturation, research gaps remain in understanding how acculturation and enculturation affect Latine mothers and adolescents. The present study contributes a better characterization of how acculturation and enculturation may function in a dyadic manner when related to language. This implies that the developmental processes of acculturation and enculturation not only happen during socialization (parent to child) but that children are also important to the acculturation of their parents. The findings from this study support that both heritage language (Spanish) and mainstream language (English) are important to the ethnic‐group‐oriented behaviors of both Latine mothers and their children. Likewise, heritage language is a significant aspect of White (mainstream) orientation for Latine youth, while the relationship between youth English and youth White orientation is only apparent when perceived conflict is lower or average. All in all, many factors should be considered when seeking to understand the dynamic between language proficiencies and ethnic‐racial orientations for Latine youth and their mothers. To conduct further research, we must broaden our investigations to include children as actors for the outcomes of their mothers. Additionally, these findings indicate that to better identify the pieces of acculturation and enculturation influential to Latine families, we must acknowledge the characteristics of their environment that nurture opportunities to explore the multi‐faceted dimensions of their identities.

## CONFLICT OF INTEREST STATEMENT

The authors have no conflict of interest to declare.

## Supporting information


Tables S1–S2

## Data Availability

The data and analysis code are not publicly available but can be made available upon request from the authors.

## Terminology

“Latine” is a term most commonly used term in Latin American Spanish speaking countries to encompass various groups that may contain various gender identities (Méndez, 2023). The term “Latinx,” while frequently used, does not contain features linguistically common in Spanish. The term Latine indicates groups from Latin America with diverse gender identities with features linguistically observed in Spanish. We acknowledge the diverse identifications of people within the Latin American community beyond the term Latine which is used throughout our article.
