# Supplementary material for: Language proficiency and ethnic‐racial orientation among Latine mother–adolescent dyads
Source: J Res Adolesc. 2024 Jun 26;34(4):1352–64. doi: 10.1111/jora.12994 (PMC11606265; doi:10.1111/jora.12994)
Supplement: Supplementary file 1 — Tables S1–S2 [file JORA-34-1352-s001.docx]

**Supplemental Materials**

**S1**

*White Orientation and Acculturation Conflict (AC) Actor-Partner Interdependence Models*

|  | English model with mother-reported AC | | English model with youth-reported AC | | Spanish model with mother-reported AC | | Spanish model with youth-reported AC | |
| --- | --- | --- | --- | --- | --- | --- | --- | --- |
| Variable | *b* | *SE* | *b* | *SE* | *b* | *SE* | *b* | *SE* |
| Predicting maternal White orientation | | | | | | | | |
| Intercept | .01 | .07 | .02 | .07 | -.02 | .07 | -.02 | .08 |
| Language proficiency (mother) | **.34***** | **.08** | **.30***** | **.08** | .05 | .08 | .06 | .08 |
| Language proficiency (youth) | **.15*** | **.07** | **.18*** | **.07** | .07 | .08 | .08 | .08 |
| AC | .11 | .07 | .09 | .07 | .07 | .07 | .09 | .08 |
| Lang prof. (mother) x AC | .08 | .08 | -.04 | .08 | -.01 | .09 | .04 | .09 |
| Lang prof. (youth) x AC | -.01 | .07 | -.01 | .07 | -.005 | .08 | -.09 | .08 |
| Maternal age | **-.24*** | **.08** | **-.23*** | **.09** | **-.32***** | **.09** | **-.31**** | **.09** |
| Youth age | -.03 | .07 | -.06 | .07 | -.01 | .08 | -.04 | .08 |
| Maternal years in the US | **.26**** | **.07** | **.28***** | **.08** | **.38***** | **.08** | **.41***** | **.08** |
| Maternal country of origin | -.03 | .07 | -.06 | .08 | -.12 | .08 | -.14^T^ | .08 |
| Youth gender | -.08 | .07 | -.09 | .07 | -.05 | .07 | -.05 | .08 |
| Predicting youth White orientation | | | | | | | | |
| Intercept | -.02 | .08 | -.05 | .08 | -.02 | .08 | -.06 | .08 |
| Language proficiency (mother) | .01 | .09 | -.03 | .09 | -.13 | .08 | **-.19*** | **.09** |
| Language proficiency (youth) | **.22*** | **.08** | **.20*** | **.08** | **.24**** | **.08** | **.28**** | **.09** |
| AC | -.01 | .08 | -.07 | .08 | .04 | .08 | .03 | .08 |
| Lang prof. (mother) x AC | -.06 | .10 | -.15 | .09 | .004 | .09 | -.15 | .10 |
| Lang prof. (youth) x AC | .05 | .08 | **-.24**** | **.08** | .05 | .08 | -.09 | .09 |
| Maternal age | .05 | .10 | .03 | .10 | .07 | .09 | .07 | .10 |
| Youth age | .12 | .08 | .12 | .08 | .11 | .08 | .11 | .08 |
| Maternal years in the US | .05 | .09 | .05 | .09 | .09 | .08 | .09 | .08 |
| Maternal country of origin | -.03 | .09 | -.12 | .09 | -.04 | .08 | -.08 | .08 |
| Youth gender | -.06 | .08 | -.08 | .08 | -.01 | .08 | .0004 | .08 |

*Note. b* = standardized regression coefficient. *SE* = standard error. Youth gender coded as 0 = female, 1 = male. Maternal country of origin coded as 0 = country other than Mexico, 1 = Mexico. **p* < .05, **p < .01, ****p* < .001.

**S2**

*Ethnic Orientation and Acculturation Conflict (AC) Actor-Partner Interdependence Models*

|  | English model with mother-reported AC | | English model with youth-reported AC | | Spanish model with mother-reported AC | | Spanish model with youth-reported AC | |
| --- | --- | --- | --- | --- | --- | --- | --- | --- |
| Variable | *b* | *SE* | *b* | *SE* | *b* | *SE* | *b* | *SE* |
| Predicting maternal ethnic orientation | | | | | | | | |
| Intercept | .02 | .08 | .03 | .08 | -.02 | .08 | .01 | .08 |
| Language proficiency (mother) | **.29**** | **.09** | **.21*** | **.09** | **.21*** | **.08** | **.25**** | **.09** |
| Language proficiency (youth) | .002 | .08 | .06 | .08 | .10 | .08 | .07 | .09 |
| AC | .12 | .08 | .03 | .08 | .06 | .08 | .03 | .08 |
| Lang prof. (mother) x AC | .005 | .09 | -.10 | .09 | .14 | .09 | .09 | .10 |
| Lang prof. (youth) x AC | -.12 | .08 | -.05 | .08 | -.003 | .08 | -.06 | .09 |
| Maternal age | .06 | .10 | .05 | .10 | -.04 | .10 | -.04 | .10 |
| Youth age | -.06 | .08 | -.06 | .08 | .02 | .08 | .01 | .08 |
| Maternal years in the US | -.08 | .08 | -.04 | .09 | .04 | .08 | .05 | .08 |
| Maternal country of origin | .03 | .08 | .02 | .09 | -.01 | .08 | -.01 | .08 |
| Youth gender | .10 | .08 | .07 | .08 | .11 | .08 | .09 | .08 |
| Predicting youth ethnic orientation | | | | | | | | |
| Intercept | -.03 | .07 | -.01 | .08 | .01 | .07 | -.01 | .08 |
| Language proficiency (mother) | -.15^T^ | .09 | -.08 | .10 | -.03 | .08 | -.08 | .08 |
| Language proficiency (youth) | **.24**** | **.08** | **.18*** | **.08** | **.42***** | **.07** | **.45***** | **.08** |
| AC | **-.27**** | **.08** | **-.27**** | **.08** | -.13^T^ | .07 | **-.18*** | **.08** |
| Lang prof. (mother) x AC | **-.30**** | **.09** | -.09 | .09 | -.01 | .08 | -.11 | .09 |
| Lang prof. (youth) x AC | -.01 | .07 | -.10 | .08 | .06 | .08 | .02 | .08 |
| Maternal age | -.14 | .09 | -.20^T^ | .10 | -.12 | .09 | -.15 | .09 |
| Youth age | -.03 | .07 | -.04 | .08 | -.01 | .07 | -.02 | .08 |
| Maternal years in the US | .08 | .08 | .03 | .09 | .10 | .08 | .08 | .08 |
| Maternal country of origin | -.11 | .08 | -.17^T^ | .09 | -.07 | .07 | -.13 | .08 |
| Youth gender | **-.19*** | **.07** | -.15^T^ | .08 | -.12 | .07 | -.09 | .08 |

*Note. b* = standardized regression coefficient. *SE* = standard error. Youth gender coded as 0 = female, 1 = male. Maternal country of origin coded as 0 = country other than Mexico, 1 = Mexico. ^T^*p* < .10, **p* < .05, **p < .01, ****p* < .001.
